# The impact of immersive virtual reality training in thyroid surgery: a prospective randomized controlled trial

**DOI:** 10.1007/s13304-025-02387-8

**Published:** 2025-08-29

**Authors:** Anestis Basios, Nikolaos Voloudakis, Stefanos Atmatzidis, Maria Velikoudi, Evangelia Bellou, Kyriakos Vamvakidis, Basileios Papaziogas, Ioannis Koutelidakis

**Affiliations:** 1 Second Surgical Department, G. Gennimatas General Hospital of Thessaloniki, Aristotle University of Thessaloniki, Ethnikis Aminis 41, 546 35 Thessaloniki, Greece; 2 Department of Endocrine Surgery, Henry Dunant Hospital Center, Athens, Greece

**Keywords:** Endocrine surgery, Virtual reality, Training, Thyroid surgery, Randomized control trial

## Abstract

Up to this date, there have been no reports on immersive virtual reality (IVR) training in thyroid surgery. The purpose of this blinded, randomized controlled trial was to determine the validity and impact of VR training in residents’ education. Nineteen general surgery residents participated in the trial comparing IVR with traditional learning, utilizing a technical textbook as a control. A stratified randomization was used to secure balance in the distribution of residents according to their experience. The examined task pertained to the surgical steps from neck incision up to the placement of the continuous intraoperative neuromonitoring electrode and was evaluated by a blinded Fellow of the European Board of Surgery (FEBS) certified endocrine surgeon. Training superiority was assessed by the outcome measures of Objective Structured Assessment of Technical Skills (OSATS) score, need for the main surgeon to intervene, verbal answers, and time to task completion. Participants completed questionnaires regarding face validity and perceived benefits of the educational process. Immersive VR group completed the task significantly faster (p = 0.012), at a mean time of 27.25 ± 3.8 vs 35.25 ± 6.5 min in the control group, and was superior in OSATS Overall score (p = 0.035), knowledge of instruments (p = 0.015) and flow of operation (p = 0.021) scores. In the dedicated questionnaire, the participants of the IVR group stated greater overall satisfaction of the educational process (p = 0.002), ease of use (p = 0.015), enjoyment (p < 0.001), repetitivity (0.001), and perceived improvement in surgical technique (0.021). The IVR group dedicated more time in training 39 ± 8.9 vs 27.5 ± 7.2 min (p = 0.014), and performed more repetitions of the educative module, 3.5 (2–5) vs 2 (1–3) (p = 0.003) than the control group. IVR training demonstrated improved outcomes in several translational technical skills acquisition over traditional learning, while yielding improved satisfaction and repetitivity for participants. *Clinical trials registration number No*: NCT06917755.

**Supplementary Information:**

The online version contains supplementary material available at 10.1007/s13304-025-02387-8.

## Introduction

Virtual and augmented reality technology is a promising educational tool that can supplement surgeons’ training curriculums using applications on different kinds of operations. Definitions of Virtual Reality (VR) or Augmented Reality (AR) were given by Miligam et al. and Muhanna for VR devices [1]. A VR device creates a fully synthetic environment in which users can be immersed. Simple properties such as gravity, time and matter can be surpassed or simulated to a certain extent [2]. Users are presented in front of a fully virtual world through a display mask. On the other hand, in augmented reality, virtual content can be directly superimposed on reality, resulting in a more reality-based experience [3]. Immersive simulator analysis and validation has been used extensively in the aviation industry and military. However, it has been limited in procedural medical education until recent years [4].

The rationale behind simulated training projects is that such devices offer a repeatable educational process which is safe for the patient. Also, many young surgeons are familiar with such applications, so they are trained in a more enjoyable and familiar way. Training cost effectiveness seems to be a significant potential advantage of VR training [5]. According to certain US studies, between 2017 and 2018 there were 25,537 residents in surgical programs and 4760 radiology residents which approximates $4.1 billion in direct training costs. Other issues include regional variables and hospital structures, educational stuff, facilities, clinical and operative duties, and salaries. Considering those metrics, a simulator that provides a surrogate to operations or diagnostics would be of considerable value for the medical community, especially in steepening the learning curve of trainees [6]. A strong boost of such applications was mandated by the COVID-19 pandemic which interrupted a significant portion of operations worldwide and as a result the whole training activity. Educators had to find novel ways of providing consistent and efficient training to ameliorate the lack of real-world training opportunities [7].

The role of “conventional” VR training in laparoscopic and robotic surgery training has been previously established in various studies and meta-analysis [8, 9]. However, “conventional” VR devices, such as laparoscopic simulators, do not apply in open surgeries, which demand the development of immersive VR applications [10]. Despite the presence of several studies investigating IVR applications in Orthopedic, Maxillofacial, Plastic or Neuro- surgery [10], up to this date, there are no studies regarding immersive virtual reality (IVR) training in thyroid surgery.

The hypothesis of this study was that IVR training would provide improved technical skill acquisition in comparison with conventional training methods, such as an endocrine technical book and instructional videos. To determine this, the Objective Structured Assessment of Technical Skills (OSATS) tool [11] was used to assess the superiority of IVR training or traditional training as the primary objective. As secondary objectives this study included the time to task completion, training time and training repetition, verbal answers on composite questions, need for chief surgeon`s intervention and need of operation cessation due to safety reasons. Furthermore, participants assessed the face validity of the educational modules and rated their experience of IVR training.

## Methods

### Participants

Nineteen general surgery residents affiliated with the 2nd Surgical Department of Aristotle University of Thessaloniki were recruited in the study protocol following institutional ethics review board approval. The study took place from 1/8/2024 to 31/1/2025. Subjects were required to have completed at least 6 months of their residency program, to be more familiar with handling of surgical instruments and operational safety protocols. None of the recruited subjects had completed the 6th year of training. The performance evaluators were Fellows of the European Board of Surgery (FEBS) certified endocrine surgeons performing ≥ 50 thyroid operations annually. Patients participating in the study provided their informed consent following extensive description of the intervention and resident involvement. Patients presenting with more challenging cases such as advanced thyroid cancer [12], recurrence/reoperation, concomitant primary hyperparathyroidism, lymph node metastasis, Grave’s disease and large goiters were excluded from the study protocol.

### Randomization

**Fig. 1:**
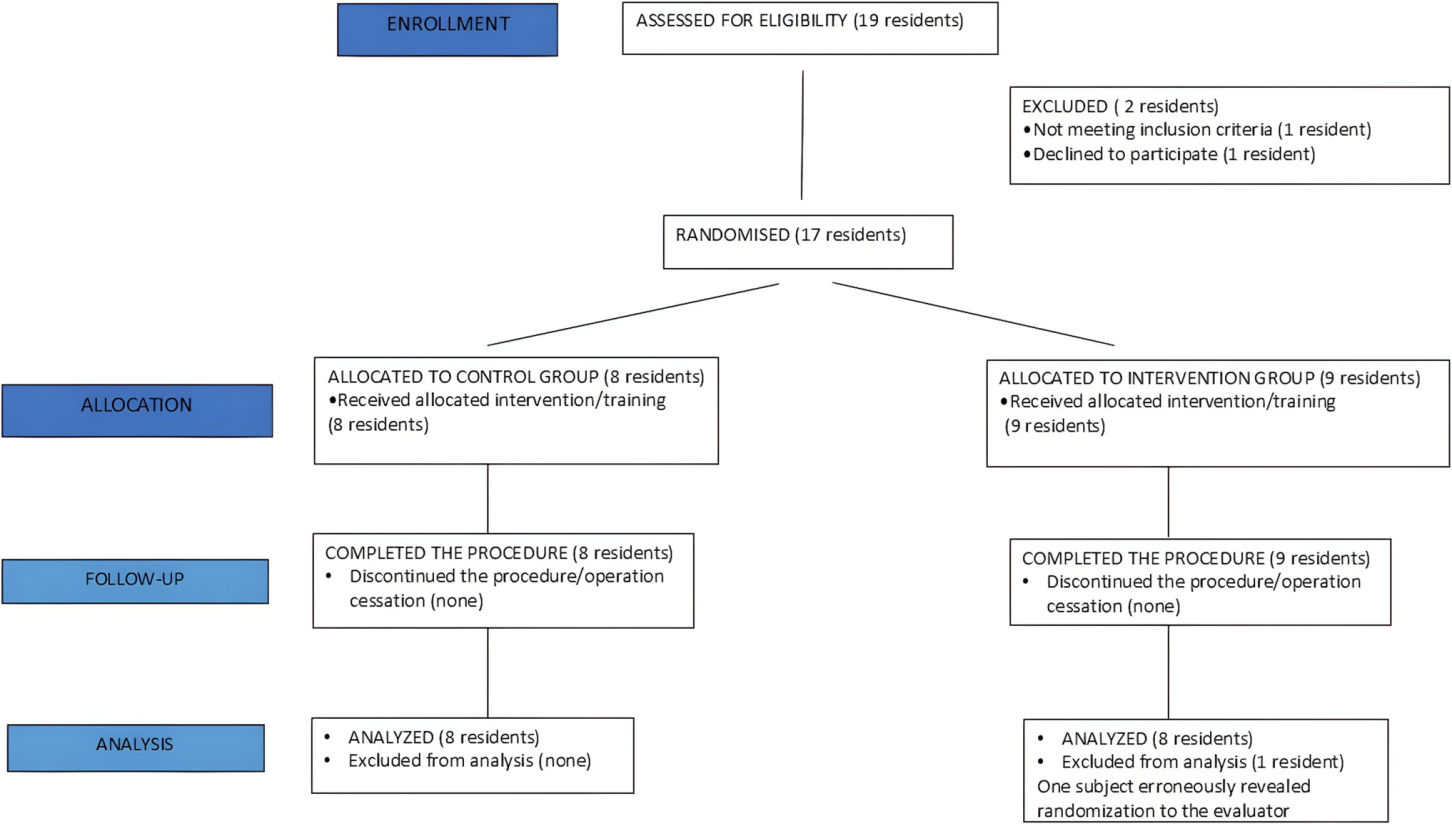
CONSORT flow diagram of randomization of participants

A block stratified randomization, according to residents’ experience level (less than two years of residency, 3–4 years, 5–6 years) was held and categorized them into equal Immersive Virtual Reality and control groups. Evaluators were also allocated based on block randomization and were blinded as to participants’ allocation group. Participants were unaware of the allocated training module/intervention until the training module initiated. Figure 1 depicts the CONSORT(Consolidated Standards of Reporting Trials) flow diagram for the study.

### Intervention

**Fig. 2:**
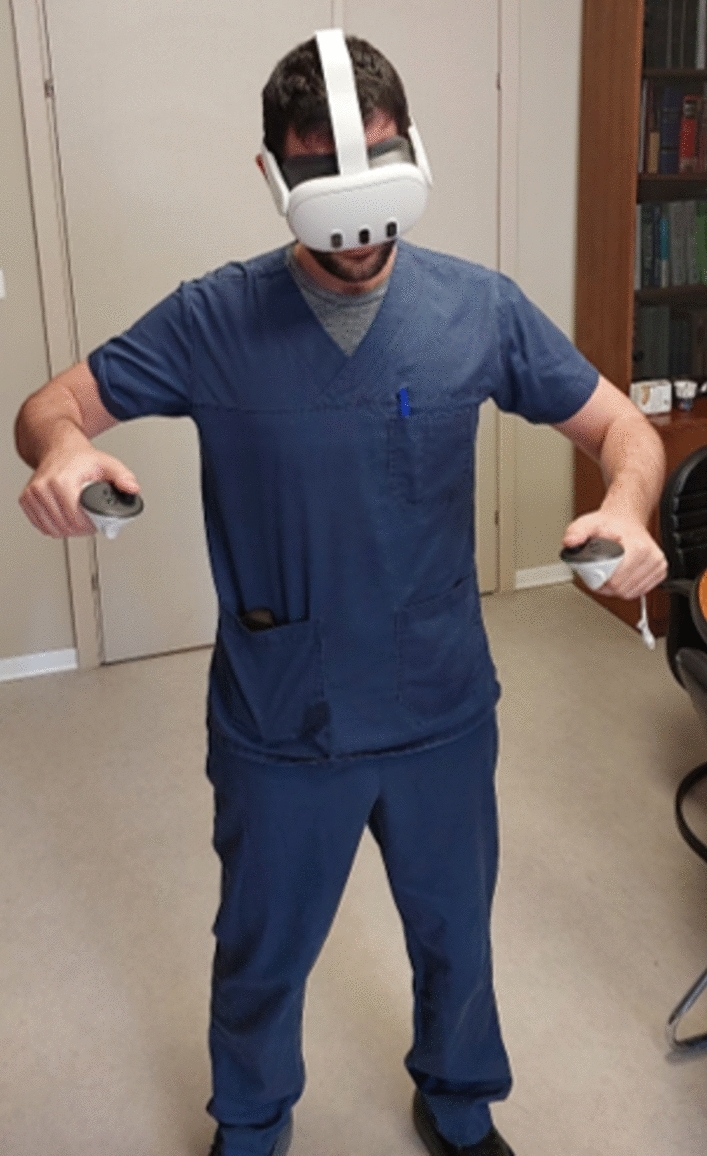
Participant in the virtual reality training group

**Fig. 3:**
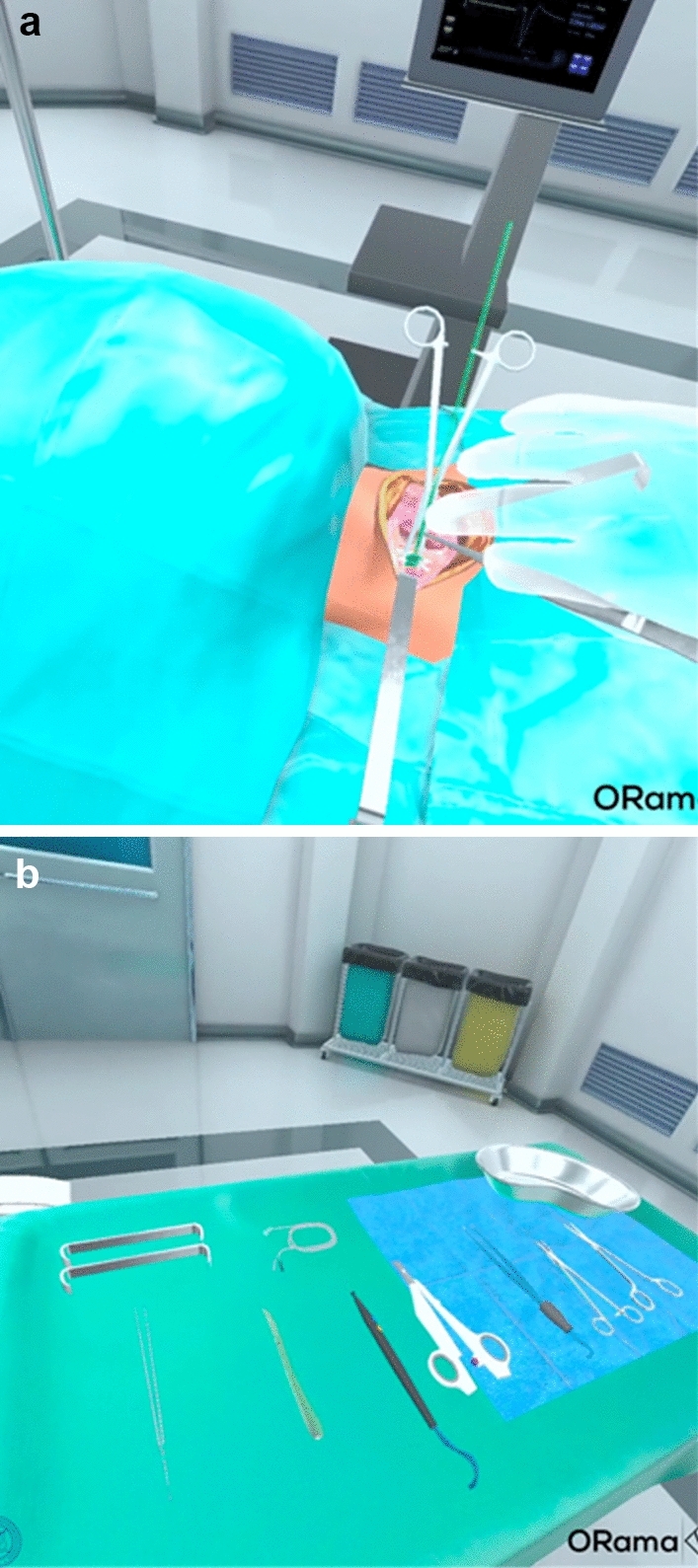
**a** Trainee’s view of the surgical field in the VR application, **b** Trainee’s view of the surgical tools available

The Immersive Virtual Reality group performed the first key steps of thyroidectomy- from neck incision to vagus nerve continuous neuromonitoring device placement in a VR environment. A MetaQuest3 VR device was used, which included head-mounted display and haptic controllers. A dedicated training application was designed by OramaVR Inc. (Geneva, Switzerland). The development of the application was commissioned and approved by the executive board of the Hellenic Society of Endocrine Glands Surgery. The application illustrated specific steps in thyroidectomy including neck incision, dissection of the subcutaneous tissue, creation of sub-platysma flaps, middle line dissection, vagus nerve dissection and placement of a continuous intraoperative neuromonitoring (C-IONM) electrode on the vagus nerve (Figs. 2 and 3). Subjects of this group had to complete the process at least once, but were deliberately left with the option to resume training as long as they wished to investigate the user`s engagement.

**Fig. 4:**
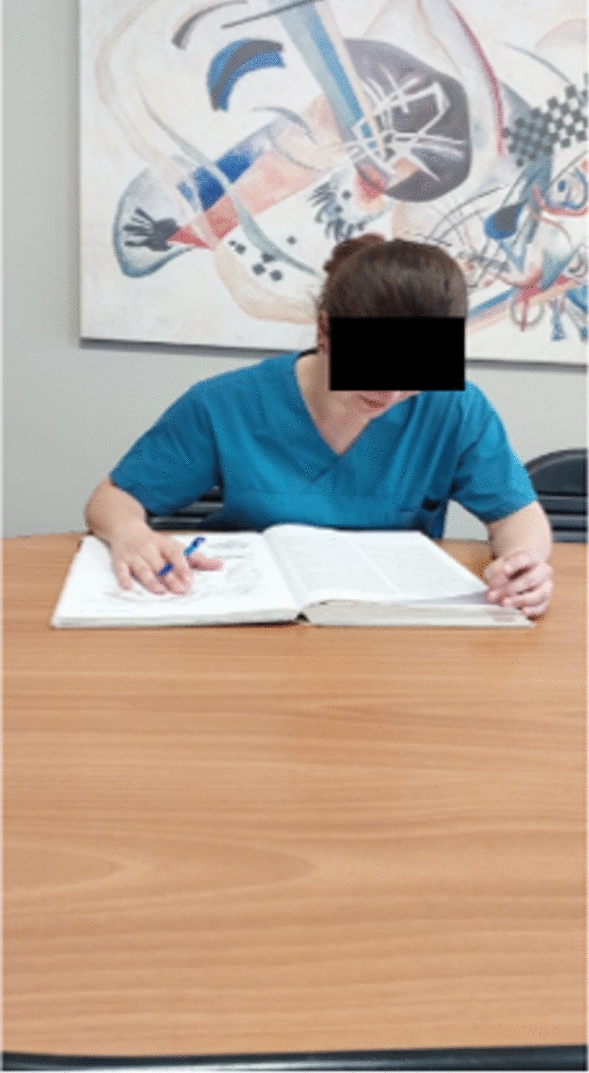
Participant during studying the selected chapter from a technical surgical book

**Fig. 5:**
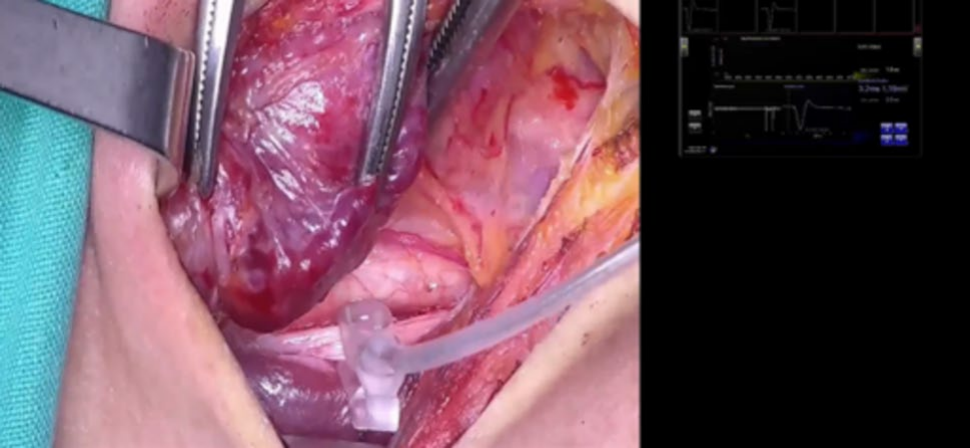
Placement of continuous intraoperative neuromonitoring electrode—snapshot from the training video of the control group

The control group was provided with a well-established technical book of endocrine surgery [13] outlining in specific chapters the first key steps of the operation (Fig. 4). As no technical book delves deeper into C-IONM electrode placement, this group was provided with an educational video of the department`s archive, especially focused on this part (Fig. 5). Control subjects also had no limit on time or repetition of their training, but had to cover the material given at least once. The training of both groups took part one day before the operation, following normal work-hours, in the department’s premises. Trainees were notified two weeks prior for the day of intervention/training and operation as to enable them to have the evening free of other obligations.

**Fig. 6:**
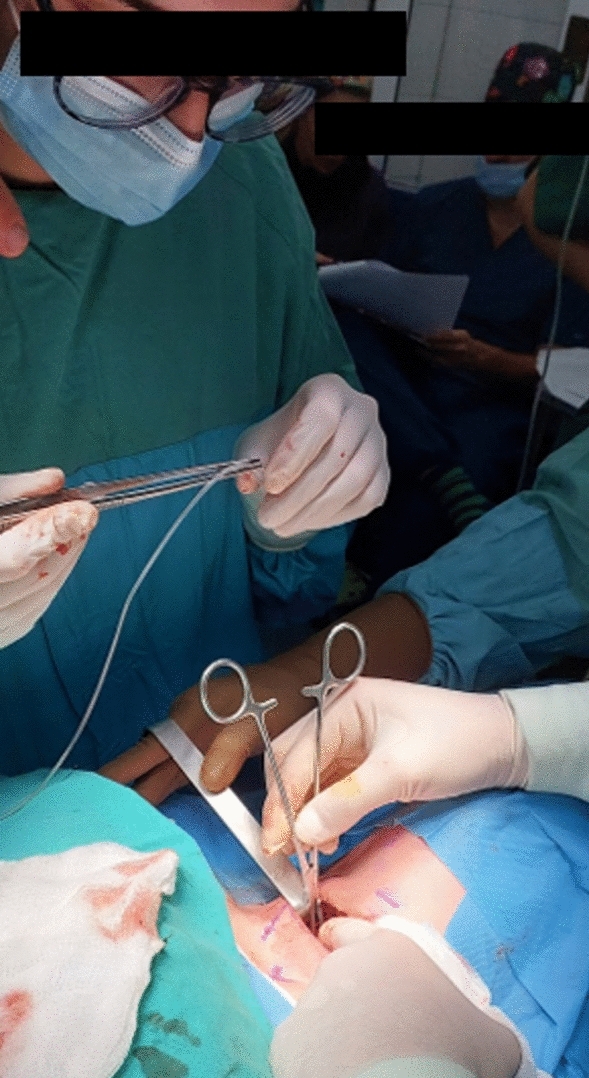
Participant during electrode placement in the operating room

Each participant, after completing the allocated intervention the previous day, performed the first steps of thyroidectomy, from neck incision to vagus C-IONM device placement (Fig. 6). The whole process was under supervision of a main operator, an experienced endocrine surgeon, who was free to intervene for safety reasons. The instances that the main operator intervened were also recorded. A blinded FEBS certified endocrine surgeon assessed the whole process and evaluated the participant with no information regarding the participant`s allocation group. The whole process could be ceased by the main operator, or the evaluator, at any time if they deemed that the safety of the patient was compromised. In addition, 5 composite technical questions were addressed to each participant regarding the anatomy of the neck and surgical steps performed, which were also evaluated.

After completion of the allocated task, vagus nerve C-IONM electrode placement, the main operator completed the thyroid surgery.

After completion of the operation, each participant evaluated the whole experience with a Likert scale questionnaire regarding face validity of the entire educational process, as well as the perceived benefits of the training module. Meanwhile, the evaluators assessed the participants performance with the OSATS score.

### Study outcomes

The primary outcome measure of the study was the OSATS (Objective Structured Assessment of Technical Skills) score to determine the superiority of VR or conventional training [11, 14, 15].

Secondary outcomes included participant`s demographics, time of task completion, answers to technical questions, a face validity questionnaire previously employed in similar protocols, as well as engagement variables of the subject with the allocated training module [6].

Since this was a study concerning real patients, two parameters were added to ensure patient safety: attending surgeon need for intervention and need of evaluation cessation due to safety reasons. Instances of attending surgeon’s intervention consisted of verbal corrections to the participant when the flow of the operation was disrupted, or the perceived resident’s action might seem excessive regarding tissue handling.

### Statistical analysis

To achieve 80% statistical power ($\beta$ = 0.02) using a 2-sided test at $\alpha$ = 0.05, a minimum of 7 participants were required for each group, based on a pilot study performed with 5 participants in each group. Randomization was performed with the dedicated application RRApp [16]. Distribution of variables was assessed with the Shapiro–Wilk test. Continuous variables were reported as mean (± standard deviation) or median (range, minimum–maximum value) depending on distribution type. Differences between groups were assessed by Student’s t or Mann–Whitney *U* test for parametric and non-parametric variables respectively. Categorical variables were analyzed using chi-square and Fisher’s exact test where appropriate. Data analysis was performed with IBM SPSS Statistics for Windows, Version 25.0 (Armonk, NY: IBM Corp). All analyses were two-tailed.

## Results

A total of 19 residents were assessed for eligibility. One resident declined to participate due to time constraints, while one resident had not completed 6 months of training. Of the 17 residents randomized, 9 were allocated in the IVR group while 8 in the control group. The results of one resident were not analyzed since he accidentally revealed his allocation group to the evaluator. Concerning demographics, in both groups there were 3 residents of less than two years of experience, 3 residents with three to four years and 2 with 5 to 6 years (*p* = 1). In the IVR group there were 5 female residents and 3 males, while in the control group 6 female residents and 2 males (*p* = 1). In both groups there were no patient complications observed, or loss of signal, up to the electrode placement by the residents.

### Outcomes

As shown in Table 1 and Supplementary Fig. 1, the IVR group significantly outperformed control group in OSATS total score (*p* = 0.035). This was mainly attributed to improved knowledge of instruments (*p* = 0.015) and flow of operation (0.021) assessment. There was a trend for improved outcomes in the rest of OSATS variables, without yielding a significant difference. The IVR group completed the designated task faster (*p* = 0.012), as shown **in **Supplementary Fig. 2. The instances that the main/attending surgeon needed to intervene and the performance of the residents in answering composite questions regarding the anatomy and technical steps did not differ between the two groups (*p* = 0.858 and *p* = 0.913 respectively). There were no cases were the evaluation needed to cease due to safety reasons (*p* = 1). Residents of the IVR group dedicated more of their free time in training with the module allocated to them (*p* = 0.014), while repeating the module more times than the control group (*p* = 0.003).

**Table 1:** Comparison of outcomes between the two groups

	Immersive VR (*n* = 8)	Control (*n* = 8)	*p*
**Training Time **(mean, SD in min)	39 ± 8.9	27.5 ± 7.2	**0.014**
**Times the trainees repeated the training module** (median, range)	3.5 (2–5)	2 (1–3)	**0.003**
**Instances of attending surgeon intervention** (mean, SD)	2.75 ± 1.3	2.88 ± 1.5	0.858
**Evaluation cessation for safety reasons**	0	0	1
**Time to task Completion** (mean, SD in min)	27.25 ± 3.8	35.25 ± 6.5	**0.012**
**Composite Questions**^a^ (median, range)	2 (1–5)	2.5 (1–4)	0.913
**OSATS**^b^** Overall score **(median, range)	24 (21–33)	19.5 (15–31)	**0.035**
Respect for Tissue	4 (3–5)	3.5 (2–5)	0.127
Type and motion	3 (2–5)	2.5 (1–4)	0.187
Instrument handling	4 (3–5)	3 (3–4)	0.441
Knowledge of instruments	4 (4–5)	2.5 (2–5)	**0.015**
Flow of operation	4 (3–5)	3 (1–4)	**0.021**
Use of assistants	3 (2–4)	2.5 (2–5)	0.496
Knowledge of specific procedure	3 (2–5)	3 (2–4)	0.35

Bold letters signify statistical difference between groups

^a^Questions related to surgical steps and common pitfalls, assessed with a Likert scale of 1 to 5, with 5 being the best score

^b^All Objective Structured Assessment of Technical Skills (OSATS) scores follow a Likert scale of 1 to 5, with 5 being the best score

Concerning the perceived benefits, in a Likert scale of 1 to 5, the participants rated the IVR training module higher in all parameters as shown in Table 2**.** For the face validity of the IVR application, it was deemed quite realistic by the participants, with lower scores concerning the realism of tissue interaction, since haptic feedback was suboptimal in the VR module. The face validity scores among younger residents were higher than their seniors, but due to a small sample size the results were inconclusive and the subgroup analysis was not included in the current manuscript.

**Table 2:** Perceived benefits and Face validity of virtual reality application

	Immersive VR (*n* = 8)	Control (*n* = 8)	*p*
*Perceived benefits*^a^			
Enjoyment	4.5 (4–5)	2.5 (2–4)	** < 0.001**
Ease of use	4 (4–5)	3 (3–4)	**0.015**
Repetitivity	4.5 (4–5)	3 (2–4)	**0.001**
Improvement in surgical technique	4.5 (4–5)	4 (2–4)	**0.021**
Overall satisfaction of educative tool	4.5 (4–5)	3 (2–4)	**0.002**
*Face validity*^a^			
Overall Realism	4 (3–5)	N/a	
Equipment Realism	4 (3–5)	N/a	
Anatomy Realism	3.5 (3–5)	N/a	
Interaction with tissues Realism	2 (2–4)	N/a	
Instrument Movement Realism	3.5 (2–5)	N/a	
Overall Similarity to Operating Room	3.5 (2–4)	N/a	

^a^All Questions assessed with a Likert scale of 1 to 5, with 5 being the best score

## Discussion

In this study, the superiority of training surgical residents with an IVR module over traditional means was shown in multiple parameters.

As early as 1993 there were suggestions of using VR devices on surgical education, but due to limited technological advancement, that was postponed until the early 00`s [17]. VR applications in the medical field are limitless. Indeed in 2002 Professor Mark Stevens at Stanford University used a VR device to simulate a patient's heart and chose the optimum approach to minimally replace the mitral valve [18]. Concerning education and training, the Work Group for Evaluation and Implementation of Simulators and Skills Training Programs of the European Association of Endoscopic Surgeons (EAES) released in 2006 a consensus guideline for validation of 6 different simulators which translated to a certain level of recommendations for each system. This act delineated the Virtual Reality training in surgery and placed specific recommendations in the field [19].

More recently, in 2015, during the Innovation, Design, and Emerging Alliances in Surgery (IDEAS) Conference, the working group identified specific elements of VR training as prerequisites for a productive implementation of VR training programs. For example, skill acquisition, flexible learner performance and rehearsal of different procedures thought to be key parameters [20].

Regarding VR training in the surgical field, there are two types commonly confused in the literature—conventional and immersive VR. “Conventional” VR is perceived as the use of a laparoscopic/endoscopic simulators, while the true/immersive VR (IVR) refers to equipment that consists at the minimum of a head-mounted device and hand controllers that enable “complete” immersion of the trainee in the virtual world[10]. Although remote access operations in thyroid surgery that could benefit from “conventional” VR gain ground, they still represent a very small portion of the total operations performed, at least in the European landscape. As such, the need to supplement residents’ and endocrine fellows’ training cannot be accommodated by conventional VR modules. Up to date, no dedicated IVR application has been developed for open thyroid surgery, as it has been observed in other fields [10]. With this in mind, the Hellenic Society of Endocrine Glands Surgery, commissioned and oversaw the creation of such an application that is inline with the key parameters dictated by the IDEAS recommendations.

In this blinded randomized study, the participating residents exhibited superior results when trained with an IVR application in contrast to traditional means. This was shown both in the overall OSATS score and in the time required for task completion. It is of note that the main task, which was the placement of a C-IONM electrode in the vagus nerve, was a process that all residents, independent of their experience level, were quite unfamiliar with. Although all operations in the department are performed with intraoperative neuromonitoring, an overwhelming percentage is done with the intermittent one due to reduced cost of expendables.

Nonetheless, the stratified randomization, categorized subjects in similar levels of experience and managed to normalize the distributed participants into equal groups. Apart from the benefits of immersion during training that might have led to superior results in technical skills during real surgery, another possible explanation of the IVR group`s performance may be the fact that IVR training was more enjoyable and easier to use. That lead to more time allocated and increased repetitions. Consequently, engagement of the subjects was high, which was exhibited in our results, and has been previously reported by similar studies. [21, 22].

Furthermore, the study was designed in such a manner as to approximate a typical preparation of a resident before scheduled surgery. The intervention/training took place after normal working hours, when in theory it was the residents’ leisure time. The engagement of the IVR training was evident, since the IVR group opted to spend significantly more time training and repeated the process several times. According to the FIRST trial published in 2016, surgical residents have flexible working programs [23]. To compensate for potential loss of surgical experience due to this problem, simulated training was proposed. Nowadays, despite working hours reduction, there is great concern concerning burnout which is estimated between 33 to 55% [24], or even higher, among surgical residents [25]. Learning time represents a great percentage of the whole working schedule of young residents. Thus, a properly designed VR module could reduce significantly the total training time, or at least render it more enjoyable.

It is indisputable that the optimal training takes place in the operating theatre[26]. However, there are several ways to steepen the learning curve. In open surgery the main surrogates are cadaveric or animal-model hands-on courses, which can be very financially demanding for a young doctor. IVR represents a viable, more economical alternative, which is projected to be pursued in the future [10]. Although IVR modules, at present, might be inferior to sophisticated cadaveric models such as SimLife® [27, 28], their development and implementation into training pathways can potentially limit the need for cadaveric courses, or prepare trainees for one.

Delving deeper, simulated training seems to be cost-effective. It is estimated that an IVR headset could be found on the market for 300–800$ and can be used multiple times by multiple doctors. On the other hand, a surgical course cost for a single resident ranges from 1000 to 1500$ [29], not including potential travel and accommodation costs. Funding of these courses could be reconsidered after the latest data of IVR vs conventional training methods, as our study exhibited. The cost-effectiveness of IVR training further increases when we incorporate the per-minute OR cost of a young surgeon to be trained properly by conventional methods [30]. Apart from limitless repetition, the IVR modules also allow for multiplayer or telementoring mode, as it all comes down to software development. As the programming cost is also the most expensive part of an IVR module, the financial burden should be shouldered by the corresponding national/international surgical societies.

### Limitations

This study has several limitations. It had a relatively small sample size, which was inevitable to maintain a homogenous sample of participants and evaluators. Residents from other hospitals could be potentially included if an adequate sample size was not achieved, but there would be no safeguard that their familiarity with thyroid operations would correspond to their year of residency. If other parameters, such as number of thyroid operations participated/assisted on, was used as a stratification criterion, that would still create a variance of their actual experience and active participation, depending on respective department’s protocols. In addition, the application used was developed only for the first steps of thyroidectomy and as a result the deducted conclusions cannot be addressed for the whole procedure. An updated version of the application is under construction which will include all steps of thyroidectomy. Furthermore, there is not a specialized rating scale of thyroid surgery performance in the English language, but OSATS score has been previously validated in other open surgeries of various specialties [31–34].

The design of our study could be improved by additional evaluators per participant to eliminate bias, but FEBS certified endocrine surgeons are limited to two in our clinic and one of them had to be the main/attending surgeon and the other the evaluator. Also, our study compared IVR with conventional methods such as reading a technical surgery book and video presentation in a selected population, but there are a lot of other training modalities (instructional courses, cadaveric dissections, etc.) that have not been tested yet in different level of subjects` experience. Thus, VR training superiority cannot be generalized and, in this developing form, could not substitute traditional learning methods but could assist effectively the whole learning process. Future research efforts should include greater sample size and updated versions of similar applications. A modern addition would be to incorporate patient`s thyroid data to IVR devices and practice on a model with individual metrics in a more realistic manner with unlimited rehearsals before surgery.

In conclusion, IVR demonstrated improved outcomes in several translational technical skills acquisition parameters over traditional learning, while yielding improved satisfaction and repetitivity for participants.

## Supplementary Information

Below is the link to the electronic supplementary material.

Supplementary file1 (PNG 78 KB)

Supplementary file2 (PNG 100 KB)

## Data Availability

The datasets generated during and/or analysed during the current study are available from the corresponding author on reasonable request.
