# Supplementary figures and images for: The impact of immersive virtual reality training in thyroid surgery: a prospective randomized controlled trial
Source: Updates Surg. 2025 Aug 29;77(8):2535–43. doi: 10.1007/s13304-025-02387-8 (PMC12630229; doi:10.1007/s13304-025-02387-8)

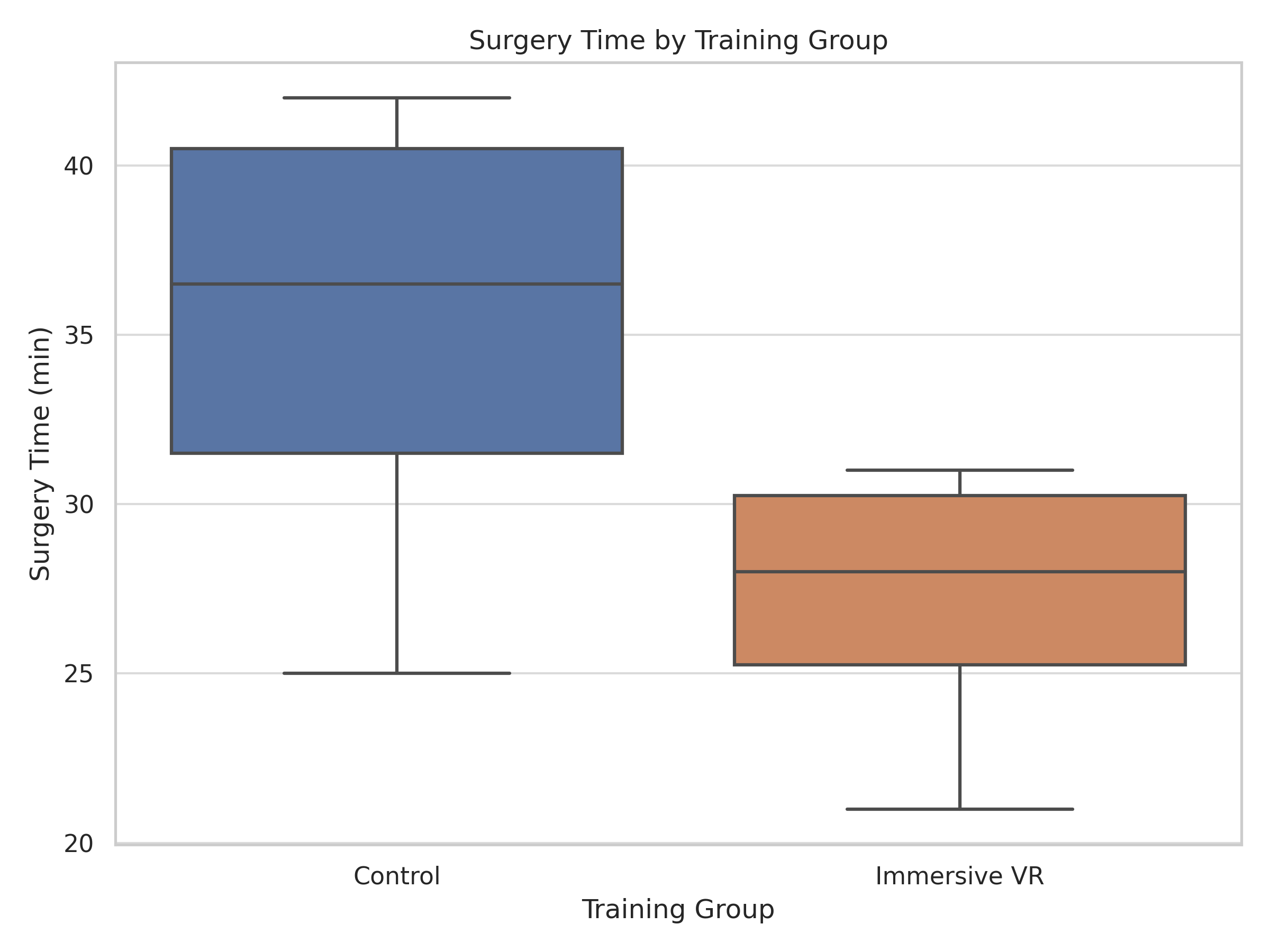

Supplement: Supplementary file 1 — Supplementary file1 (PNG 78 KB) [file 13304_2025_2387_MOESM1_ESM.png]

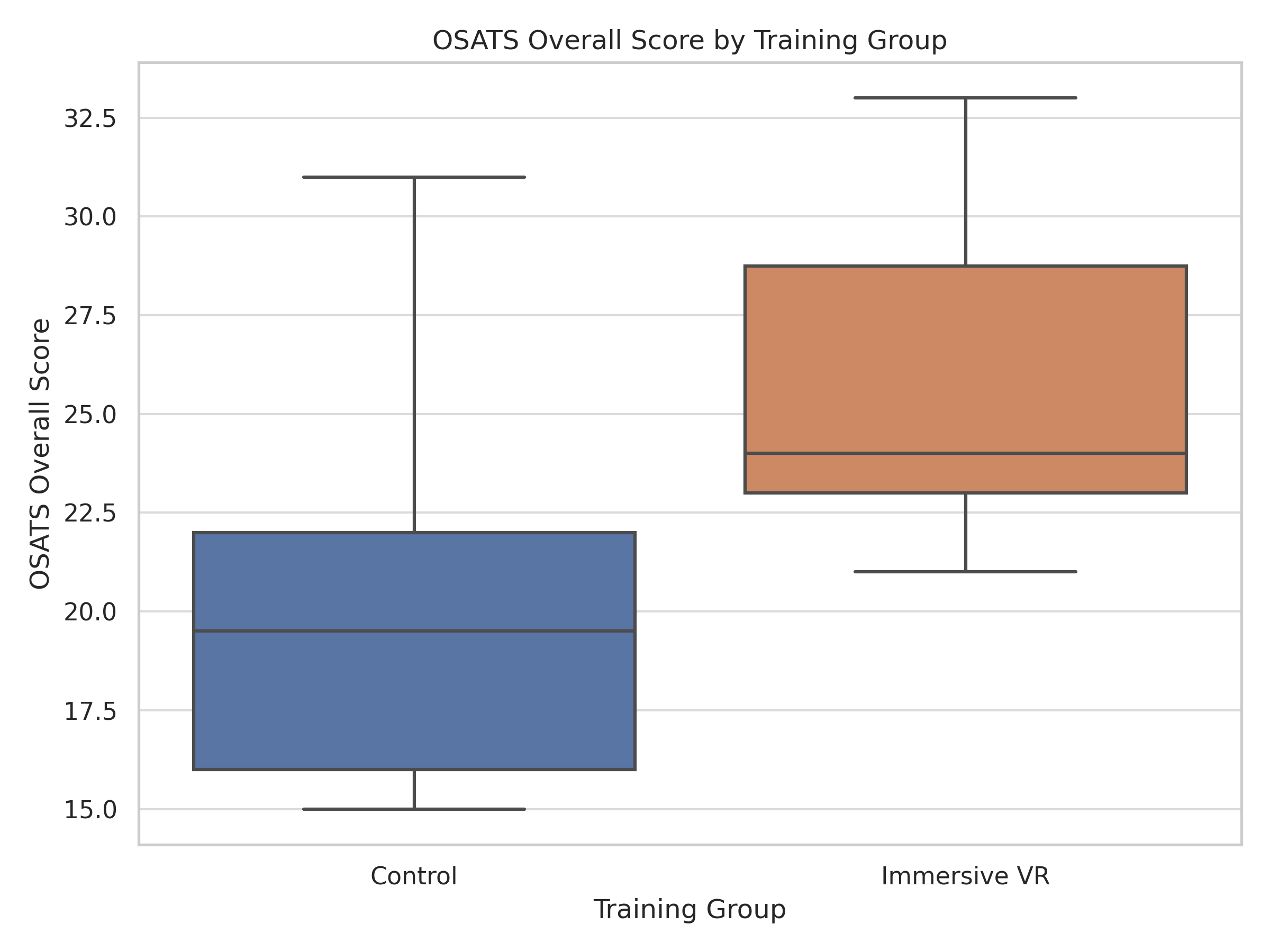

Supplement: Supplementary file 2 — Supplementary file2 (PNG 100 KB) [file 13304_2025_2387_MOESM2_ESM.png]
